# α‑Helical Peptides Encoded in Collagen Exhibit Antimicrobial Activity with Low Cytotoxicity

**DOI:** 10.1021/acs.jnatprod.5c01318

**Published:** 2026-01-13

**Authors:** Scott A. Jarmusch, Taj Muhammad, Ulf Göransson, Adam A. Strömstedt

**Affiliations:** Pharmacognosy, Department of Pharmaceutical Biosciences, 8097 Uppsala University, Box 591, 751 24 Uppsala, Sweden

## Abstract

Endogenous antimicrobial peptides (AMPs) derived from host proteins represent a largely underexplored class of natural products tied to innate immunity. Here, we investigated collagen proteins as a source of latent α-helical AMPs encoded within nonfibrous extracellular matrix domains. Using a targeted *in silico* approach, verified collagen sequences were mined and prioritized based on secondary structure and three essential physicochemical properties: net charge, Boman index, and hydrophobic moment, yielding 107 predicted α-helical AMP candidates. The highest ranked peptides were synthesized and experimentally evaluated alongside benchmark AMPs and peptides prioritized by machine learning-based prediction tools. Three collagen-derived peptides identified by the targeted physicochemical approach exhibited broad-spectrum bioactivity against bacterial and fungal pathogens with minimum inhibitory concentrations comparable to those of LL-37 and melittin. In contrast, peptides ranked highly by machine learning predictors showed reduced or no activity. Collagen-derived peptides disrupted bacterial mimicking lipid membranes yet displayed markedly reduced cytotoxicity toward human cells, maintaining high viability at concentrations well above their antimicrobial MICs. These findings demonstrate that nonfibrous domains of extracellular matrix collagens constitute a previously underexplored reservoir of endogenous antimicrobial peptides with favorable biocompatibility, expanding the natural product space of host defense peptides and identifying collagen-derived AMPs as promising scaffolds for future antimicrobial discovery.

## Graphical abstract

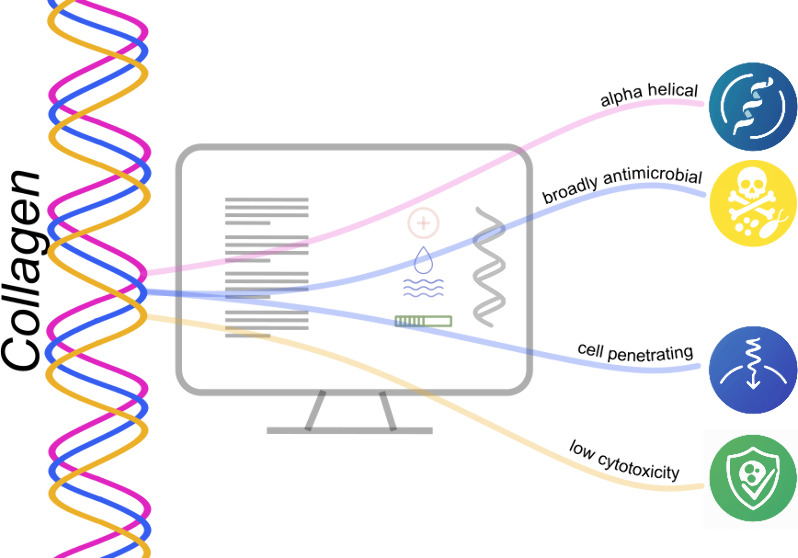

While drug discovery efforts continue, a worrying drop-off in the discovery of chemical novelty has appeared.^1^ Antimicrobial peptides (AMPs) look to be one promising ‘new’ source that the global health community could rely upon.^2–6^ A distinct advantage of AMPs is the low and slow resistance development observed due to the targeting of bacterial and fungal membranes by more general physicochemical properties, instead of specific affinity for intracellular components^3,7–9^ that more conventional antibiotics target.^10,11^ α-Helical AMPs like cathelicidins (e.g., LL-37) have proven to be an exciting source of therapeutic AMPs derived from vertebrates.^12^

AMPs play vital roles in animal health;^13^ mammals become more susceptible to infectious diseases when cathelicidin expression is inhibited.^14–17^ These bioactive fragments, often termed “cryptides,” arise through physiological proteolysis of abundant host proteins and represent an emerging class of natural products with distinct biological functions.^18^ Similar endogenous peptides have been described from hemoglobin,^19^ casein,^20^ and elastin,^21^ yet the potential of collagen as a reservoir of antimicrobial cryptides remains largely unexplored. Collagen also exhibits an antimicrobial effect upon proteolytic degradation^22^ and exploration of the α3 strand of collagen VI in bovine cornea showed α-helical, cationic regions that exhibited *in vivo* antimicrobial and wound healing activities, similar to LL-37.^23^ Despite this compelling evidence, collagen-derived AMPs have been investigated primarily on a case-by-case basis, and it remains unclear whether antimicrobial cryptides are a general feature of extracellular matrix collagens or are a property restricted to specific collagen subtypes. No systematic cross-collagen analysis has yet been undertaken to assess the prevalence, structural characteristics, or biological properties of AMPs encoded within collagen proteins. Given the extracellular localization, continuous proteolytic turnover, and evolutionary conservation of collagens, a broader evaluation of their potential role as sources of endogenous AMPs is warranted.

Here, we combined *in silico* and experimental approaches to explore the latent antimicrobial potential encoded within collagen proteins. Verified collagen sequences from UniProt were mined using the AMPA prediction tool^202^ followed by secondary structure prediction with PEP-FOLD3 ^203^ to prioritize α-helical peptide regions. Candidate peptides were ranked using a targeted physicochemical scoring system based on three key properties (net charge, Boman index, and hydrophobic moment) selected to minimize redundancy while capturing essential features of membrane-active α-helical AMPs. From 107 predicted collagen-derived α-helical peptides, the top five were synthesized and evaluated for antimicrobial activity, membrane permeabilization, and cytotoxicity. Predictions from this reduced-complexity approach were directly compared with those from commonly used machine-learning-based AMP predictors.

## Results and Discussion

### Nonfibrous Regions of Collagen Proteins Encode Latent Antimicrobial Peptide Regions

*In silico* prediction of AMPs has become increasingly common due to the cost-effective nature of DNA sequencing and the growing availability of protein sequence data. Accordingly, both classic support vector machine (SVM) models and more recent deep learning-based predictors have been widely applied to AMP discovery.^5^ In the present study, we focused on SVM-based approaches that are well suited to linear peptides, reflecting the largely linear primary structure of collagen proteins and their lack of additional cross-linkages. Specifically, three SVM-based AMP prediction tools were utilized: AMPA, CAMP_R3_, and iAMPpred.

AMPA employs an antimicrobial propensity scale derived from high-throughput screening of the AMP bactenicin 2A,^24^ whereas CAMP_R3_ and iAMPpred are machine learning-based predictors trained on curated AMP data sets. For both CAMP_R3_ and iAMPpred, we selected SVM-based algorithms, as these have previously been identified as the best-performing classifiers within each platform.^25^ While deep-learning approaches have shown promise in AMP prediction, we prioritized models that offer interpretability and are compatible with systematic screening of host-derived protein sequences. As an initial benchmark, collagen VI (α3 chain) was used to compare the performance of the three AMP prediction tools, as five antimicrobial peptides have previously been experimentally identified from this protein.^22^ When the full-length collagen VI (α3) sequence was analyzed using AMPA, nine potential AMP regions were identified and two of these overlapped with peptides reported by Abdillahi et al.^23^ In contrast, CAMP_R3_ and iAMPpred do not permit full-length protein input, and therefore the five previously described collagen VI-derived AMPs were individually analyzed; CAMP_R3_ classified all five peptides as antimicrobial, whereas iAMPpred identified only one as antimicrobial. In addition, a truncated version of the previously described SFV33 peptide^22^ was detected using AMPA. This peptide (TRK18) was synthesized as a collagen-derived AMP control, as all prediction tools evaluated classified it as antimicrobial. Based on its ability to process full-length protein sequences and its higher yield of predicted AMP regions per protein, AMPA was selected for systematic screening of all verified collagen proteins available in UniProt (n = 104).

Because collagen proteins are ubiquitous across the animal kingdom and are a constant component of the extracellular matrix (ECM), the UniProt data set encompassed a broad taxonomic range, including collagens from humans, vertebrates, and invertebrates such as insects. Initial AMPA screening identified 232 potential antimicrobial regions. After the removal of redundant sequences, 200 peptides were retained for secondary structure analysis. To capture sufficient sequence context and allow for the formation of secondary structural elements, predicted AMP regions were extended by ± 10–15 residues prior to structure prediction. These approximately 50 amino acid long sequences were subjected to secondary structure prediction using PEP-FOLD3.^26^ Based on the resulting simulations, 107 peptides were predicted to adopt α-helical conformations and were carried forward for physicochemical property analysis (Figure 1). Mapping these predicted α-helical peptides to collagen subtypes revealed a clear enrichment within nonfibrous regions of ECM-associated collagens. Collagen VI contained the highest number of putative AMP sequences, followed by collagen IV and fibril-associated collagens with interrupted triple helices (FACIT collagens). In contrast, collagen V contained relatively few predicted AMP regions, and very few putative AMPs were identified in non-ECM collagens, such as type I collagen. Notably, no predicted AMP sequences were found within fibrous collagen regions, consistent with the strict amino acid sequence requirements necessary for triple-helix formation and the corresponding absence of the α-helical secondary structure.

**Figure 1:**
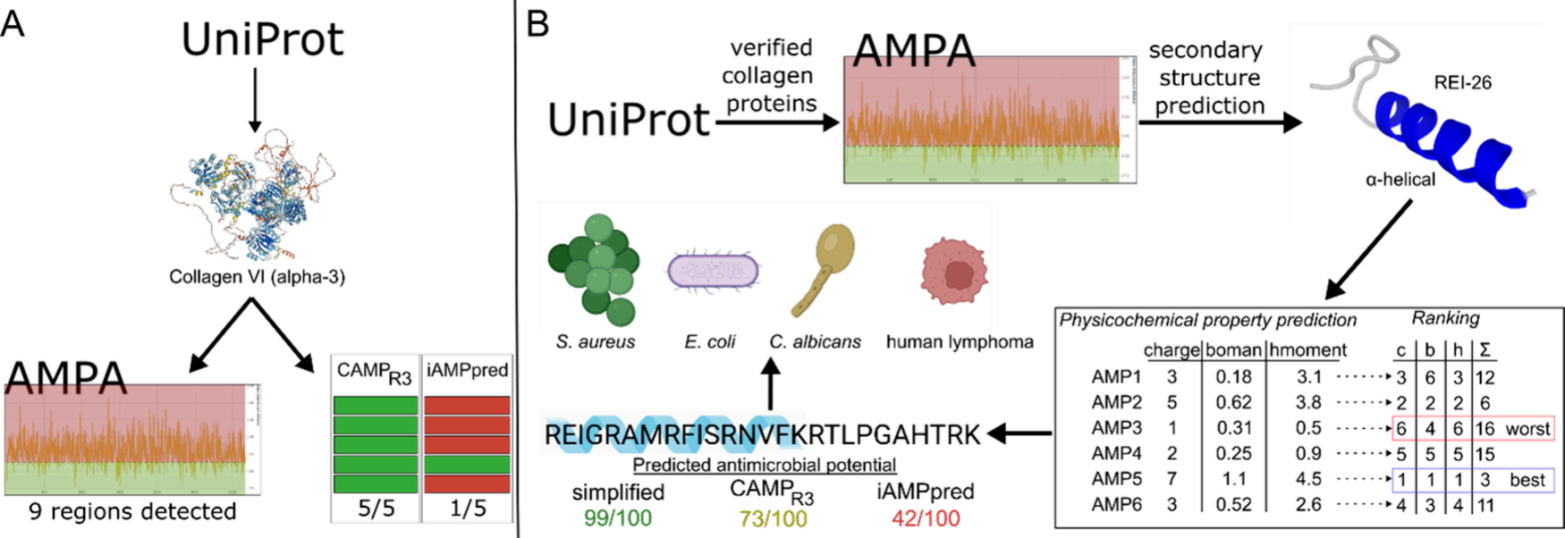
Collagen AMP discovery workflow. (A) Initial testing completed using Collagen VI (alpha-3) and ranked in the three AMP prediction tools. AMPA was selected because it predicted all of the AMPs from the previous work on collagen VI (alpha-3) and additionally predicted more potential AMPs (B) The full workflow is representative of the targeted prediction method subsequently used throughout this study: peptides identified by AMPA were filtered by secondary structure predictions for α-helical peptides, and then their charge, Boman index, and hydrophobic moment were predicted, summed, and ranked. These targeted predictions were then compared to the two predominant online bioinformatics tools, CAMP_R3_ and iAMPpred, and candidate peptides were chosen for synthesis and biological testing. Image was created in BioRender.

Taken together, these results indicate that predicted AMPs are not randomly distributed across collagen proteins but are preferentially encoded within flexible, nonfibrous regions of ECM-associated collagens. This distribution is consistent with previous observations that collagen-derived antimicrobial activity arises from nonfibrillar domains and suggests that ECM collagens represent a structured and biologically plausible reservoir of latent antimicrobial peptides.

### Physicochemical Properties Define an AMP-Enriched Prediction Space

There are a number of physicochemical properties that are tied to helical AMPs that make predictions more robust,^3,27–29^ however, three seem to be more essential for their accurate prediction: charge, boman index and hydrophobic moment. All are major physicochemical factors for higher selectivity toward bacterial membranes rather than eukaryotic cells.^30^ It is well established that charge (typically ≥ + 2) is an integral parameter to bind to a typical anionic phospholipid bilayer,^2,31^ thus being tied directly to cationic peptide mode of action. Boman index, or the potential protein binding, indicates a peptide’s potential to interact with membranes or other proteins. The index value is computed from the mean solubility based on the amino acid sequence, where lower values represent more hydrophobic properties of the peptide as a whole. AMPs tend to exhibit negative indices, indicating they unlikely interact with other proteins besides the membrane.^32^ Lastly, the hydrophobic moment (hmoment) is a quantitative measure of directional amphiphilicity, an integral aspect of cationic peptides in order to bind to as well as tunnel through membranes (<0.2 is considered good). These three properties were chosen as our essential physicochemical factors influencing AMP quality with the aim to reduce any redundancies or overlap with other properties. Additional prediction tools like CAMP_R3_ and iAMPpred utilize further physicochemical factors and coefficients as prediction parameters but can result in overlapping parameters (*e.g.*, charge and isoelectric point) that tilt predictions toward certain peptide properties.

Using the output from AMPA predictions, physicochemical properties were calculated using the R package ‘Peptides’. Beyond the 107 collagen-derived predicted AMPs, LL-37 and 5 AMPs from Abdihalli et al.,^22^ 368 α-helical AMPs from the Antimicrobial Peptide Database,^33^ and 50 AMPs and 50 non-AMPs from the ‘Peptides’ data training set were all included into physiochemical property predictions. Every peptide was ranked from 1 to 581 in each property and then summed (lower score = better AMP), allowing for a targeted AMP ranking based on the three physical properties described above (Figure 1). To visualize how collagen-derived peptides compare with known α-helical AMPs across key physicochemical parameters, predicted peptides were projected into pairwise 2D spaces defined by net charge, hydrophobic moment, and Boman index (Figure 2A). Synthesized collagen-derived peptides cluster within the same region of physicochemical space as LL-37. To further contextualize these predictions, α-helical AMP quality scores were compared between putative collagen-derived peptides and α-helical peptides from the Antimicrobial Peptide Database, revealing that the highest-ranking collagen peptides fall within the upper range of α-helical AMP quality values (Figure 2B).

**Figure 2:**
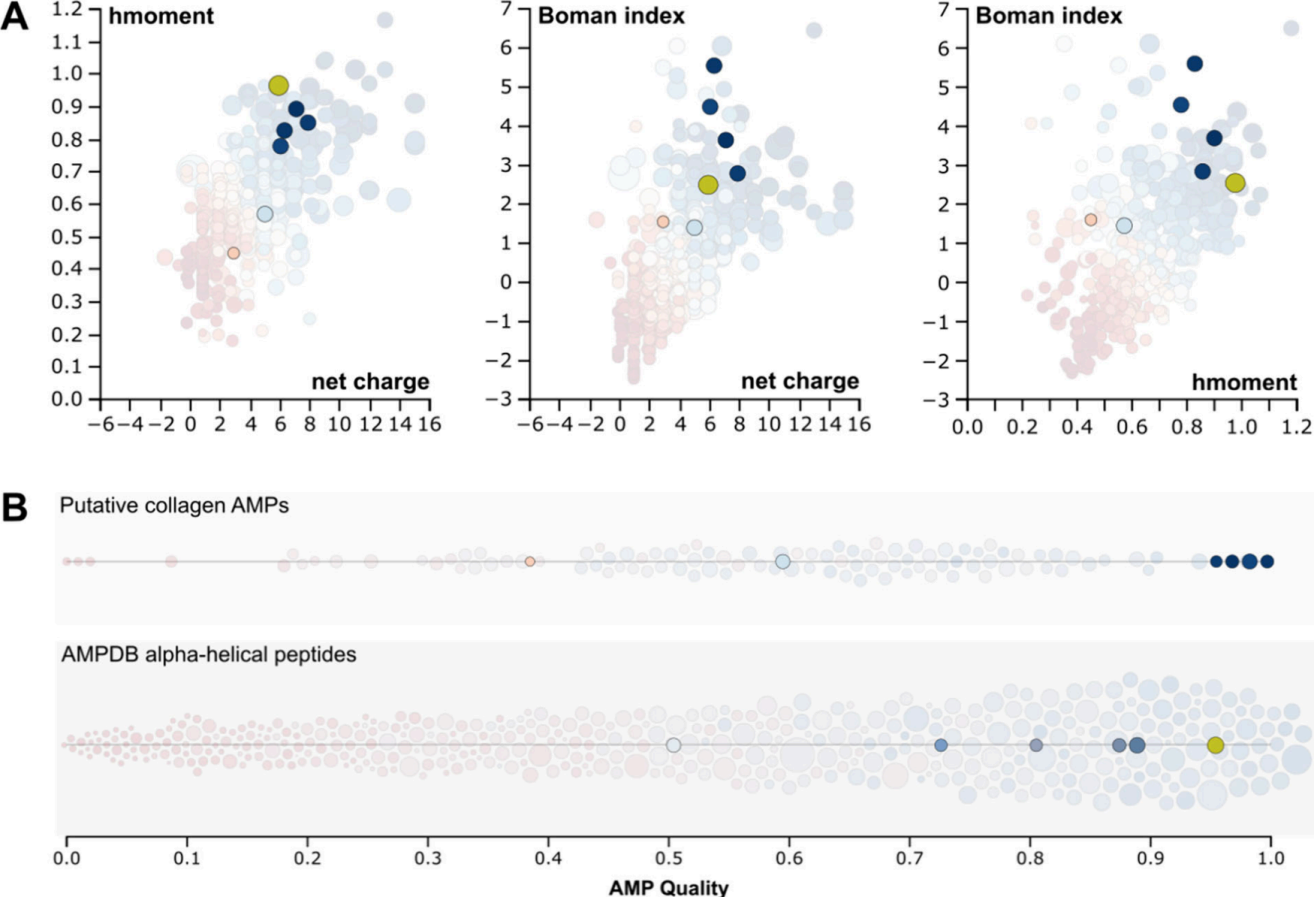
Physicochemical landscape and quality ranking of predicted collagen-derived antimicrobial peptides. Color scale indicates α-helical AMP quality (0 = red, 1 = blue). (A) Pairwise two-dimensional projections of the net charge, hydrophobic moment, and Boman index for AMPDB peptides (transparent), synthesized collagen peptides (opaque), and LL-37 (yellow). (B) Distribution of AMP quality scores for (top) putative collagen-derived peptides (transparent) and sythesized peptides (opaque) and (bottom) α-helical peptides from the Antimicrobial Peptide Database (transparent), LL-37 (yellow) and five collagen-derived AMPs discovered by Abdillahi et al.,^22^ highlighting the relative enrichment of high-scoring collagen-derived candidates. Circle size reflects peptide molecular weight. Benchmark peptides in this study include LL-37 and the five collagen-derived AMPs discovered by Abdillahi et al.^22^

### Targeted Predictions Identify Collagen-Derived Peptides with Enriched Antimicrobial Activity

Based on the composite rankings derived from charge, Boman index, and hydrophobic moment, the top five predicted antimicrobial peptides were selected for solid-phase peptide synthesis: REI-26, LEL-28, TRR-26, LRS-21, and SPE-22 (Table 1). All five peptides ranked higher than the benchmark AMP LL-37 according to the summed physicochemical scores. To contextualize these rankings, we additionally evaluated all collagen-derived peptides identified by AMPA using CAMP_R3_ and iAMPpred. While some overlap was observed between CAMP_R3_ and iAMPpred predictions, their highest-ranked peptides differed markedly from those prioritized by the targeted physicochemical approach (Table 1). Notably, peptides ranked highly by the targeted approach spanned a wide range of predicted AMP quality in CAMP_R3_ and iAMPpred, with LRS-21 classified as a non-AMP by CAMP_R3_.

**Table 1:** Top 5 Putative Antimicrobial Peptides from Collagen Proteins, as Predicted by Three Separate Tools^a^

Predictor	AMPA + ‘Peptides’ Rank	CAMP_R3_ Rank	iAMPpred Rank	Peptide sequence	CAMP_R3_ class	CAMP_R3_ Score	iAMPpred score
AMPA + ‘Peptides’	98.97	73.04	41.74	REIG​RAMR​FISR​NVFK​RTLP​GAHTRK	AMP	0.719	0.272
	98.28	92.17	48.70	LELH​VRRE​LHRL​RRDV​SHLQ​LTRR​QQRR	AMP	0.901	0.303
	97.25	91.30	30.43	TRR​RVRR​TSRL​VAKR​ALLL​CILL​YCT	AMP	0.893	0.176
	96.91	31.30	90.43	LRSP​GISR​FRRK​IAKR​SIKTL	NAMP	0.268	0.818
	95.70	54.78	22.61	SPEY​PEHM​RWKR​SLSR​KAKRKP	AMP	0.502	0.133
CAMP_R__3_	38.49	99.13	73.04	GPEN​FSKM​KMFM​KNL​VSKS	AMP	0.993	0.585
	59.45	98.26	97.39	GFDILLGLDVNKKVKKRIQLSPKKIKGYEV	AMP	0.989	0.985
	44.50	96.52	98.26	IGALK​QAVK​NLKW​IAGG​THTG	AMP	0.979	0.993
	18.21	93.91	99.13	LLAA​SPVA​SKGCV​CKGK​GQCL​CAGTK	AMP	0.964	0.995
	56.36	93.04	93.91	KALA​LGAL​QNIR​YRGG​NTRT​GKAL​TFIK	AMP	0.946	0.871
iAMPpred	18.21	93.91	99.13	LLAA​SPVASKGCV​CKGK​GQCL​CAGTK	AMP	0.964	0.995
	44.50	96.52	98.26	IGALK​QAV​KNLKW​IAGG​THTG	AMP	0.979	0.993
	59.45	98.26	97.39	GFDIL​LGLD​VNKK​VKKR​IQLSP​KKIK​GYEV	AMP	0.989	0.985
	53.09	68.70	95.65	LQDA​KKIFF​GSFH​KVHIA​VGHS​KVRL​YVDCRK	AMP	0.664	0.956
	56.36	93.04	93.91	KALA​LGAL​QNIR​YRGG​NTRT​GKAL​TFIK	AMP	0.946	0.871

a Rankings are scaled to 100, where 100 is the most antimicrobial.

Conversely, peptides ranked highest by CAMP_R3_ and iAMPpred generally ranked in the middle to lower range of the targeted prediction system. To directly evaluate the predictive performance of these alternative tools, two additional peptides were selected for synthesis: GPE-19, the top-ranked peptide predicted by CAMP_R3_, and GFD-30, which ranked second and third by CAMP_R3_ and iAMPpred, respectively. Three benchmark peptides were included for comparison: LL-37, melittin, and TFK-18, a novel truncated collagen VI-derived AMP derived from the previously described SFV33.^23^ Finally, to test whether high charge alone was sufficient to confer antimicrobial activity, a peptide derived from the fibrous region of collagen VI (α3) with high net positive charge (GEK-25) was included as a predicted non-AMP control. All synthesized collagen-derived peptides and benchmark controls were evaluated for antimicrobial activity using a two-step microdilution assay optimized for antimicrobial peptides. Activity was tested against three phylogenetically and structurally distinct human pathogens: *Escherichia coli* (Gram-negative), *Staphylococcus aureus* (Gram-positive), and *Candida albicans* (fungal). Minimum inhibitory concentrations (MICs), reported as mean values, are shown in Table 2 and Figure 3.

**Table 2:** Synthesized Collagen-Derived Peptides, Their Sources, and the Nomenclature Used in This Study^a^

ID	Organism	Source	Peptide name	Sequence	*E. coli* MIC	*S. aureus* MIC	*C. albicans* MIC
Predicted AMPs	*Homo sapien*	Col6a6	REI-26	REIGR​AMRF​ISRNV​FKRT​LPGA​HTRK	5	2.5	1.25
	*Drosophila melanogaster*	Col4 CG42342	LEL-28	LELH​VRREL​HRLR​RDVS​HLQL​TRRQ​QRR	40	20.625	2.5
	*Danio rerio*	Col6a6	TRR-26	TRRR​VRRTS​RLVA​KRA​LLLCIL​LYCT	3.75	2.5	1.875
	*Gallus gallus*	Col9a1	LRS-21	LRSPG​ISRFR​RKIA​KRSI​KTL	3.75	3.75	3.125
	*Gallus gallus*	Col12a1	SPE-22	SPEY​PEHM​RWKR​SLSR​KAKRKP	30	22.5	3.75
	*Homo sapien*	Col21a1	GFD-30	GFDIL​LGLD​VNKK​VKKR​IQLSP​KKIK​GYEV	10	3.75	6.25
	*Mus musculus*	Col6a6	GPE-19	GPEN​FSKM​KMFM​KNLV​SKS	>40	>40	>40
	*Homo sapien*	Col6a3	*TFK-18	SFVAR​NTF​KRVRN​GFLM​RKVA​VFF​SNTPT​RASP	3.75	2.5	1.875
Known AMPs	*Homo sapien*	*-*	LL-37	LLGD​FFRKS​KEKIG​KEFK​RIVQR​IKDF​LRNL​VPRTES	1.25	1.875	3.75
	*Apis mellifera*	-	melittin	GIGA​VLKV​LTTGL​PALIS​WIKR​KRQQ	1.25	2.5	1.875
Predicted non-AMP	*Homo sapien*	Col6a3	GEK-25	GEKG​NPGR​RGDK​GPRG​EKGE​RGDVG	>40	>40	>40

a An example of source nomenclature is Col6a6 abbreviates for collagen type VI, alpha-6 chain. All MICs reported in this table are in units of μM. *Indicates this peptide is a truncated form of a the previously described AMP SFV33. The truncation is visible in the sequence column.

**Figure 3:**
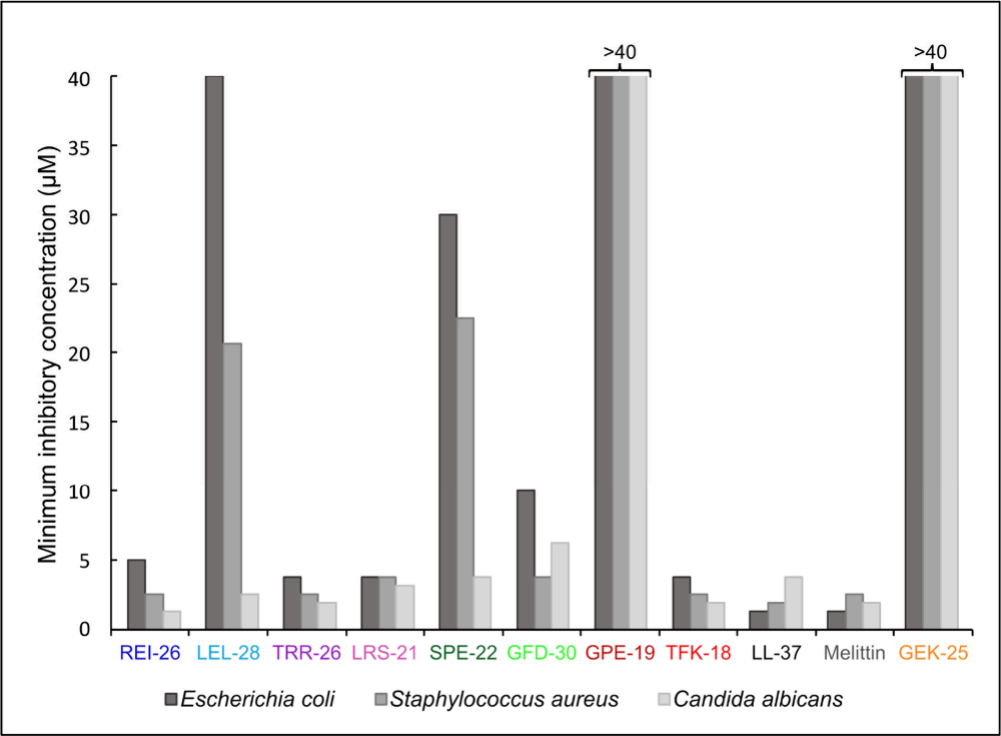
Antimicrobial activity summarized from the microdilution assay as mean minimum inhibitory concentrations (MICs) of the collagen-derived predicted AMPs and a predicted non-AMP (GEK-25), and two benchmark AMPs (LL-37, melittin).

Among the peptides prioritized by the targeted prediction approach, three exhibited the strongest and broadest antimicrobial activity: REI-26 (5, 2.5, and 1.25 μM), LRS-21 (3.75, 3.75, and 3.125 μM), and TRR-26 (3.75, 2.5, and 1.875 μM) against *E. coli*, *S. aureus*, and *C. albicans*, respectively. These MIC values were comparable to those of the benchmark peptides LL-37 (1.25, 1.875, and 3.75 μM), melittin (1.25, 2.5, and 1.875 μM), and TFK-18 (3.75, 2.5, and 1.875 μM). GFD-30 also exhibited broad antimicrobial activity but required higher concentrations to reach MIC values (10, 3.75, and 6.25 μM), indicating reduced potency relative to the top-performing peptides identified by the targeted approach. In contrast, LEL-28 and SPE-22 displayed weak activity against *E. coli* (40 and 30 μM, respectively), moderate activity against *S. aureus* (20.625 and 22.5 μM), and comparatively strong antifungal activity against *C. albicans* (2.5 and 3.75 μM). GPE-19, despite being the top-ranked peptide predicted by CAMP_R3_, exhibited no detectable antimicrobial activity at concentrations of up to 40 μM against any microorganism tested. Similarly, GEK-25, derived from the fibrous region of collagen VI, showed no antimicrobial activity, supporting the prediction that a high charge alone is insufficient to confer AMP activity in the absence of appropriate secondary structure and amphiphilicity.

As expected for membrane-active peptides, most predicted AMPs exhibited activity across multiple microorganisms within the 1–40 μM concentration range tested. However, the stark inactivity of GPE-19 is particularly informative, as it demonstrates that high-ranking predictions from machine learning-based tools do not necessarily translate into biological activity, especially for peptides that deviate from known AMP scaffolds. When antimicrobial potency was ranked by summing MIC values across all three organisms, the peptides followed the order: melittin > LL-37 > TRR-26 > TFK-18 > REI-26 > LRS-21 > GFD-30 ≫ SPE-22 > LEL-28 ≫ GPE-19 = GEK-25. Notably, three of the five peptides prioritized by the targeted physicochemical approach exhibited greater overall antimicrobial potency than the top-ranked peptide predicted by CAMP_R3_, and all five outperformed the second- and third-ranked peptides predicted by CAMP_R3_ and iAMPpred.

To assess the novelty of the identified peptides, sequence similarity searches were performed against publicly available antimicrobial peptide databases, including the Antimicrobial Peptide Database (APD) and DRAMP. No close sequence matches were identified among the >20,000 curated AMP entries, indicating that the collagen-derived peptides described here represent distinct AMP scaffolds rather than variants of previously reported peptides. Together, these results demonstrate that a targeted physicochemical prediction strategy enriches for biologically active, structurally novel AMPs encoded within collagen proteins, while also highlighting limitations of machine learning-based predictors when applied to endogenous host-derived sequences.

### Collagen-Derived Peptides Induce Membrane Permeabilization Consistent with α-Helical AMP Activity

Most α-helical AMPs exert their antimicrobial effects by compromising the integrity of the microbial cytoplasmic membrane, typically through pore formation or less-defined membrane perforation. One established method for probing this mechanism is monitoring peptide-induced permeabilization of artificial lipid membranes. Accordingly, membrane-disruptive activity of the collagen-derived peptides was assessed using large unilamellar liposomes composed of *E. coli* polar lipid extracts and loaded with a self-quenching fluorescent marker.

Benchmark α-helical AMPs LL-37 and melittin, which are well characterized for their pore-forming activity in bacterial-mimicking membranes, were included as positive controls. Liposome leakage was monitored over time, and the percentage of leakage after 45 min of incubation for each peptide concentration is shown in Figure 4. Each peptide concentration was tested independently, with peptides not added cumulatively.

**Figure 4:**
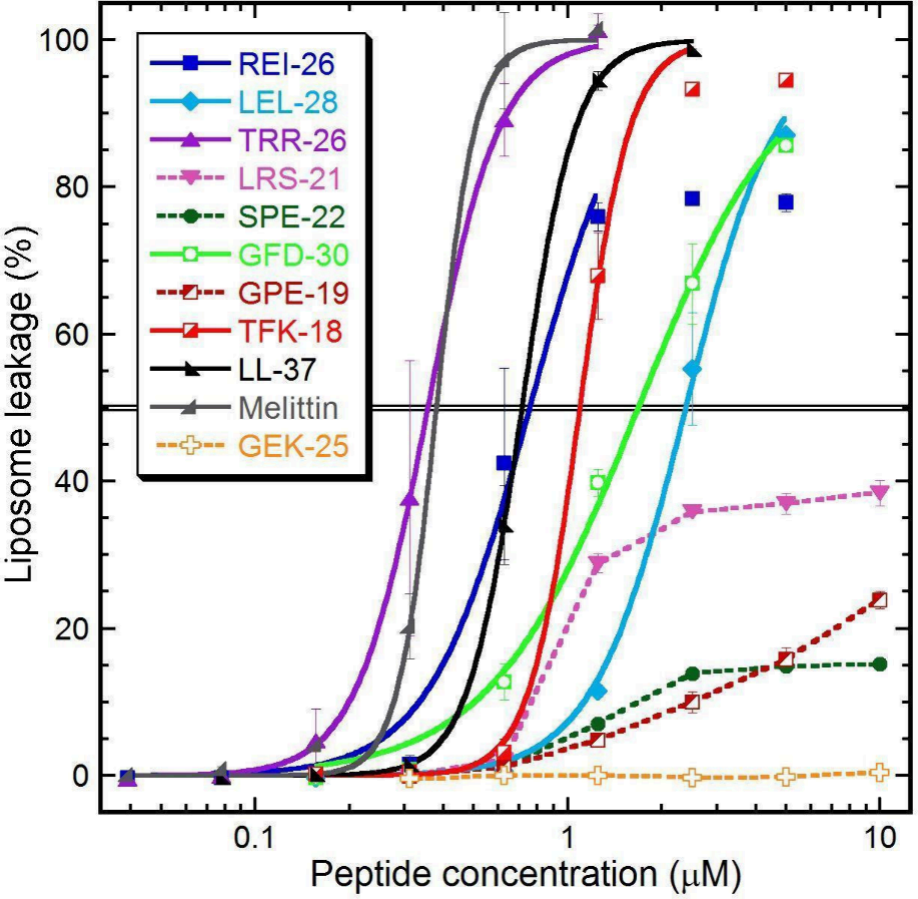
Leakage generated on liposomes manufactured from extracted *E. coli* phospholipids represents a generic microbial cytoplasmic membrane. Each mark represents the amount of leakage reached (triplicate mean with standard deviations) after 45 min of peptide incubation, at which point leakage had largely subsided. Dose–response fitting (solid line) generating EC50 in μM was done for REI-26 (0.76), LEL-28 (2.4), TRR-26 (0.36), GFD-30 (1.7), TKF-18 (1.1), LL-37 (0.71), and melittin (0.38). Other peptides did not reach an EC50 within the concentration range (dashed lines).

Among the collagen-derived peptides, the highest-ranked predictions, REI-26 and TRR-26, produced the strongest membrane permeabilization, reaching EC_50_ values comparable to those of LL-37 and melittin, respectively. TFK-18 and GFD-30 showed slightly reduced membrane-disruptive potency, followed by that of LEL-28. In contrast, GPE-19 had only marginal effects on liposome integrity, and the predicted non-AMP GEK-25 produced no detectable leakage across the tested concentration range. Overall, membrane permeabilization activity broadly correlated with antimicrobial potency observed in the microdilution assay, supporting membrane disruption as a primary mechanism of action for the active collagen-derived peptides. However, deviations from classical sigmoidal dose–response behavior were observed for certain peptides. In particular, LRS-21—and to a lesser extent SPE-22—displayed limited maximal leakage, plateauing at approximately 40% and 15%, respectively, despite exhibiting measurable antimicrobial activity. For LRS-21, the onset of leakage occurred at concentrations similar to those required for antimicrobial activity and to those observed for GFD-30, yet the extent of leakage did not increase proportionally with the concentration beyond a defined threshold.

Such behavior is not uncommon among α-helical AMPs and may reflect alternative modes of membrane interaction, including transient pore formation, saturation of membrane binding sites, or preferential association with already disrupted or fragmented liposomes. Similar plateauing behavior was also observed for REI-26, which stabilized at approximately 80% leakage, and for TFK-18, which reached near-complete leakage (∼95%). In contrast, TRR-26 induced apparent self-quenching of the fluorescent marker at higher concentrations, resulting in a concentration-dependent signal exceeding 100% leakage above 1.25 μM. This effect is consistent with the localized accumulation of carboxyfluorescein as counterions in the presence of certain cationic peptides and has been reported previously in similar assays.^200^ Importantly, this behavior was not observed for the other peptides within the tested concentration range. The EC_50_ values obtained for the benchmark peptides LL-37 and melittin were consistent with previously reported values using the same experimental setup, supporting the reliability of the assay. Taken together, these results indicate that the active collagen-derived peptides exhibit membrane-disruptive behavior characteristic of α-helical AMPs while also highlighting mechanistic heterogeneity that likely contributes to differences in antimicrobial potency and selectivity.

### Collagen-Derived Antimicrobial Peptides Exhibit Low Cytotoxicity toward Human Cells

Cationic α-helical AMPs frequently display cytotoxicity toward mammalian cells due to their ability to interact with weakly negatively charged membranes, resulting in a narrow therapeutic window that has limited their clinical translation. Accordingly, the evaluation of cytotoxicity is a critical component in assessing the therapeutic potential of newly identified AMPs. To assess the biocompatibility of collagen-derived peptides, cytotoxicity was evaluated using a fluorometric microculture cytotoxicity assay (FMCA) on human lymphoma cell line U-937 GTB.

Overall, collagen-derived AMPs exhibited markedly higher cell viability compared with the benchmark peptides LL-37 and melittin across the tested concentration range (Figure 5). As expected, melittin produced the strongest concentration-dependent decrease in cell viability, consistent with its well-documented cytolytic activity as the principal component of bee venom.^201^ LL-37 also showed substantial cytotoxicity at elevated concentrations, followed by the truncated collagen-derived peptide TFK-18. In contrast, the majority of collagen-derived peptides identified in this study showed little to no reduction in cell viability, even at concentrations substantially exceeding their antimicrobial minimum inhibitory concentrations. Only at the highest concentration tested (80 μM) was a modest reduction in viability observed for TRR-26 and for the predicted non-AMP control GEK-25. Notably, REI-26 and TRR-26 exhibited antimicrobial activity and membrane-disruptive behavior comparable to LL-37 and melittin, yet maintained 90–100% cell viability at concentrations up to 40 μM. This dissociation between antimicrobial potency and cytotoxicity is uncommon among α-helical AMPs and represents a defining feature of the collagen-derived peptides described here.

**Figure 5:**
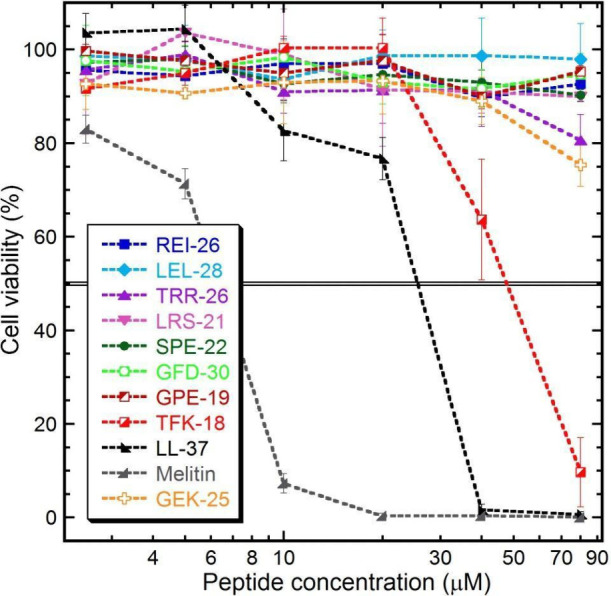
Cytotoxicity on human lymphoma cells (U-937 GTB) measured using FMCA. Peptide concentration was tested from 2.5 to 80 μM, and each mark represents the triplicate reading of the survival index, which is defined as the fluorescence in the experimental wells, expressed as a percentage of that in the control wells after the fluorescence of the blanks had been subtracted from both the experimental and the control readings.

Importantly, previous studies identifying antimicrobial peptides derived from collagen focused primarily on antimicrobial efficacy and in vivo activity and did not include systematic cytotoxicity profiling.^22,23^ As a result, whether collagen-derived AMPs possess improved host-cell compatibility relative to classical α-helical AMPs has remained unclear. The present results demonstrate that collagen-derived AMPs can combine potent, broad-spectrum antimicrobial activity with substantially reduced cytotoxicity, suggesting that low mammalian cell toxicity may be an inherent property of certain collagen-encoded cryptides rather than a consequence of sequence optimization. This observation is particularly notable given the endogenous origin and extracellular localization of collagen proteins, which are subject to continuous proteolytic turnover in tissues.^31^ It is plausible that evolutionary constraints acting on ECM-derived peptides favor antimicrobial activity while minimizing host cell damage, thereby contributing to a more favorable therapeutic index. Together, these findings identify collagen-derived AMPs as a distinct subclass of α-helical antimicrobial peptides with an unusually advantageous balance between antimicrobial potency and mammalian cell compatibility.

## Conclusions

In this study, we systematically explored collagen proteins as a source of endogenous antimicrobial peptides using a targeted physicochemical prediction strategy focused on α-helical peptides. Screening of verified collagen sequences revealed that putative antimicrobial regions are preferentially encoded within nonfibrous domains of extracellular matrix collagens, particularly collagen VI and collagen IV, rather than within rigid fibrillar regions. Experimental validation demonstrated that several of the highest-ranked collagen-derived peptides exhibit broad-spectrum antimicrobial activity comparable to benchmark α-helical AMPs, while peptides prioritized by machine learning-based predictors but not by the targeted approach showed reduced or absent activity. Together, these results indicate that ECM collagens represent a structured and previously underexplored reservoir of latent antimicrobial peptides and that targeted physicochemical filtering can be enriched for biologically active candidates within this space.

A defining feature of the collagen-derived AMPs identified here is their markedly reduced cytotoxicity toward human cells compared with classical α-helical AMPs such as LL-37 and melittin. Despite exhibiting comparable antimicrobial potency and membrane-disruptive behavior, several collagen-derived peptides maintained high mammalian cell viability at concentrations well above their minimum inhibitory concentrations. Previous studies of collagen-derived AMPs have largely focused on antimicrobial efficacy and in vivo activity without systematic evaluation of host-cell toxicity. The present findings therefore establish low cytotoxicity as a key and distinguishing property of certain collagen-encoded cryptides, suggesting that this balance between antimicrobial activity and host compatibility may be intrinsically linked to their endogenous origin and extracellular localization.

More broadly, this work highlights the value of focusing on essential, largely independent physicochemical parameters to guide AMP discovery from host proteins rather than relying exclusively on machine-learning models trained on existing AMP databases. While such models are effective at identifying peptides similar to known AMP scaffolds, they may be less suited to uncovering structurally novel, endogenous peptides such as those encoded in collagen. By demonstrating that collagen-derived α-helical peptides combine antimicrobial potency with favorable biocompatibility, this study positions ECM proteins as a promising foundation for future in silico and experimental efforts aimed at expanding the repertoire of host-derived antimicrobial peptides and identifying new AMP scaffolds with improved therapeutic potential.

## Experimental Section

### General Experimental Procedures

Fmoc-protected amino acids were obtained from PepChem (Tübingen, Germany) or Iris Biotech (Marktredwitz, Germany). Dichloromethane (DCM), N,N-dimethylformamide (DMF), and HPLC-grade acetonitrile were purchased from Thermo Fisher Scientific or Saveen & Werner. Diisopropylethylamine (DIPEA), piperidine, trifluoroacetic acid (TFA), trimethylsilylisopropane (TIPS), formic acid, phosphate-buffered saline (PBS), RPMI 1640 medium, and tryptic soy broth (TSB) were obtained from Sigma–Aldrich unless otherwise stated. Peptide synthesis was performed on a CEM Liberty 1 automated microwave-assisted peptide synthesizer using standard Fmoc/tBu solid-phase peptide synthesis protocols. Semipreparative reverse-phase high-performance liquid chromatography (RP-HPLC) purification was carried out on a Shimadzu LC-20AD system. Analytical RP-HPLC was performed using a Phenomenex Jupiter C18 column (5 μm, 300 Å, 250 × 4.6 mm) with UV detection at 215 nm. Peptide identity was confirmed by UPLC–MS using a Waters Xevo G2-XS mass spectrometer coupled to an Acquity UPLC BEH C18 column (1.7 μm, 130 Å). Fluorescence measurements for liposome leakage and cytotoxicity assays were performed using a Thermo Scientific Varioskan Flash plate reader. Peptide concentrations were determined using a Direct Detect spectrophotometer. All peptides used in biological assays were of >95% purity as determined by analytical RP-HPLC.

### In Silico AMP Identification and Physicochemical Analysis

Verified collagen protein sequences were retrieved from UniProt (n = 107) and screened for putative antimicrobial regions by using the AMPA prediction tool. Identified regions were extended by 10–15 amino acids on each side prior to secondary structure prediction. Secondary structure predictions were performed using PEP-FOLD3, and peptides predicted to adopt α-helical conformations were selected for further analysis. Candidate peptides with lengths between 18 and 30 amino acids were retained. Physicochemical properties including net charge, Boman index, and hydrophobic moment were calculated using the R package ‘Peptides’. Each peptide was ranked individually for each property, and the summed ranks were used to generate a composite physicochemical score. Additional antimicrobial peptide classification was performed using CAMPR3 and iAMPpred with the default support vector machine settings. Visualization of peptides in physicochemical space was performed using RAWGraphs 2.0.

### Peptide Synthesis and Characterization

TFK-18 and LL-37 were synthesized in-house by Fmoc/tBu solid-phase peptide synthesis on a CEM Liberty 1 automated microwave-assisted peptide synthesizer. Tentagel Rink-K Amide and Fmoc-Ser­(tBu)-Novasyn TGT resins were used as the solid supports. Peptides were assembled on a 0.1 mmol scale using a 5-fold molar excess of Fmoc-protected amino acids activated with HBTU in the presence of DIPEA (10 equiv). Following chain assembly, peptides were cleaved from the resin using a TFA/TIPS/H_2_O (95:2.5:2.5) cleavage mixture. Crude peptides were precipitated with cold diethyl ether, collected by centrifugation, dissolved in aqueous acetonitrile, and lyophilized. Melittin was obtained commercially (Sigma–Aldrich) and was used after purification. All other peptides were purchased from Proteogenix (Schiltigheim, France). Purchased and in-house synthesized peptides were purified by semipreparative RP-HPLC using a linear gradient of acetonitrile in water containing 0.05% TFA. Peptide purity (>95%) was confirmed by analytical RP-HPLC, and peptide identities were verified by UPLC–MS. Peptide concentrations were determined using a Direct Detect spectrophotometer.

### Minimum Inhibitory Concentration Assay

The antimicrobial activities of the peptides were evaluated using an AMP-adapted minimum inhibitory concentration (MIC) protocol called the two-step microdilution assay.^33^ *Escherichia coli* ATCC 25922, and *Staphylococcus aureus* ATCC 29213, as well as one fungal strain *Candida albicans* ATCC 90028, were used for minimum inhibitory determination (MIC). All of the microbial strains derived from clinical wound infections were obtained from the Department of Clinical Bacteriology at Lund University Hospital, Sweden. The microbes were grown overnight in 3% tryptic soy broth (TSB) at 37 °C to mid logarithmic phase. The microbial suspension was washed twice with 10 mM Tris buffer (set to pH 7.4 at 37 °C). 96-well plates (U-shaped, untreated polystyrene) were prepared with peptide 2-fold serial dilutions in a tris-buffer. The microbial culture density was quantified by OD_600_, diluted in Tris buffer, and 50,000 cells were aliquoted to each well in the 96-well plate to a final 100 μL volume. After 1 h of aerobic incubation at 37 °C, 5 μL of 20% (w/v) TSB medium was added to each well and the plates were incubated at 37 °C for another 16–18 h. The MIC was defined as the lowest peptide concentration that fully inhibited visible bacterial growth within the indicated time. The resulting MIC values are mean values from triplicate independent experiments. The deviations between independent experiments did not exceed one dilution factor.

### Bacterial Liposome Leakage

The liposome leakage assay was performed as previously described^34^ using *Escherichia coli* polar lipid extract as the membrane lipid source. Lipid films were prepared in round-bottom flasks, dried under reduced pressure, and rehydrated at 55 °C in 10 mM Tris buffer containing 100 mM 5(6)-carboxyfluorescein. Multilamellar vesicles were reduced by repeated extrusion through 100 nm polycarbonate membranes, and unencapsulated dye was removed by gel filtration. Peptide-induced membrane permeabilization was assessed in 96-well plates using 2-fold serial peptide dilutions in Tris buffer. Liposomes were added to a final lipid concentration of 10 μM in a total volume of 200 μL. Plates were equilibrated to 37 °C prior to liposome addition, and the fluorescence was monitored for 45 min. Triton X-100 was used to define 100% leakage. Results are reported as the percentage of total leakage after the subtraction of baseline fluorescence. Data represent the mean ± standard deviation of three independent experiments. EC_50_ values, when applicable, were calculated by nonlinear regression using sigmoidal dose–response curves with leakage constrained between 0 and 100%.

### Cytotoxicity Assay

Cytotoxicity was evaluated using a fluorometric microculture cytotoxicity assay (FMCA) with the human lymphoma cell line U-937 GTB.^35,36^ Cells were maintained in RPMI 1640 medium supplemented with 10% heat-inactivated fetal bovine serum, 2 mM glutamine, 50 μg/mL streptomycin, and 60 μg/mL penicillin and cultured under standard conditions (37 °C, 5% CO_2_). Peptides were prepared as 2-fold serial dilutions in 96-well plates, and 2 × 10^4^ cells were added per well in a total volume of 200 μL. Plates were incubated for 72 h, after which cells were washed with phosphate-buffered saline. Fluorescein diacetate (10 μg/mL) was added to each well, and fluorescence was measured after 40 min (excitation 485 nm, emission 538 nm). Cell viability was expressed as survival index (SI), defined as fluorescence in peptide-treated wells relative to untreated control wells after subtraction of background fluorescence. Data represent the mean values from three independent experiments.

## †

Department of Biotechnology and Biomedicine, Technical University of Denmark, Kongens Lyngby 2800, Denmark
